# Regression analyses of the data sets for the analysis of decomposition error in discrete-time open tandem queues

**DOI:** 10.1016/j.dib.2022.108640

**Published:** 2022-09-24

**Authors:** Christoph Jacobi, Kai Furmans

**Affiliations:** Karlsruhe Institute of Technology (KIT), Institute for Material Handling and Logistics (IFL), Gotthard-Franz-Str. 8, 76131 Karlsruhe, Germany

**Keywords:** Multiple linear regression, Quantile regression, ANOVA, Waiting time

## Abstract

The data sets and regression models presented here are related to the article “Point and interval estimation of decomposition error in discrete-time open tandem queues” [1]. The data sets are the first to analyze the approximation quality of the discrete-time decomposition approach and contain independent and dependent (explanatory) variables for the analysis of decomposition error, which were obtained using discrete-time queueing models and discrete-event simulation. Independent variables are the utilization parameters of the queues, and variability parameters of the service and arrival processes. Dependent variables are decomposition error with respect to the expected value and 95-percentile of the waiting time distribution at the downstream queue. This article presents multiple linear regression and quantile regression to explain the variance of the dependent variables for tandem queues with equal traffic intensity at both queues and for tandem queues with downstream bottlenecks, respectively.

**Specifications Table**

**Table:** 

Subject	Mathematical Modelling
Specific subject area	Discrete-time queueing theory
Type of data	Table, Chart
How the data were acquired	Data were acquired using discrete-time queueing theory and discrete-event simulation. For a given parametrization of the tandem queue (arrival and service rates, and variability parameters) we computed the expected value and the 95th-percentile of waiting time using both methods. Decomposition error is the relative divergence between the results obtained with simulation and discrete-time queueing theory, respectively [1].
Data format	Analyzed, Filtered
Description of data collection	The expected values of inter-arrival and service times (that is, the flow parameters) have been varied in the range [1.0, 30.0], the variability parameters have been varied in the range [0.1, 3.0]. The external arrival process was Poisson and the service times at both stations were gamma-distributed.
Data source location	• Institution: Karlsruhe Institute of Technology (KIT) • City/Town/Region: Karlsruhe • Country: Germany
Data accessibility	Repository name: Repository KITopen, hosted by the Karlsruhe Institute of Technology (KIT), Karlsruhe, Germany Data identification number: https://doi.org/10.5445/IR/1000148460 Direct URL to data (research data): https://bwdatadiss.kit.edu/dataset/461#headingFileList
Related research article	C. Jacobi, K. Furmans, Point and interval estimation of decomposition error in discrete-time open tandem queues, Operations Research Letters 50(5) (2022) 529-535, https://doi.org/10.1016/j.orl.2022.07.009.

## Value of the Data

• Decomposition approaches for open queuing networks are known to yield approximate results for the analysis of non-renewal downstream arrival processes [1]. The data sets described and analyzed in this article are the first to investigate the approximation quality (that is, decomposition error) of the discrete-time decomposition approach. Decomposition error is the relative divergence between the waiting time (expected value and 95th-percentile), obtained with simulation and discrete-time queueing theory, respectively [1].
• The regression analyses reveal statistically significant correlations between the variability and utilization parameters of the tandem queue and decomposition error. This suggests that decomposition error can be efficiently estimated with only the input parameters of the tandem queue at hand. Researchers deploying decomposition approaches can use the data and regression models to alert severe decomposition errors with high forecasting accuracy.
• This data may also help for the design of experiments of the analysis of the approximation quality of decomposition approaches for other network typologies (e.g. stochastic splits and merges), as well as for the validation of exact decomposition methods.

## 1 Data Description

The data repository supplied with this article contains raw data for the analysis of decomposition error in discrete-time open tandem queues. The data is formatted for the computation and validation of point and interval estimates for decomposition error as well as for the analysis of decomposition error in bottleneck queues.

The repository contains two folders:

“01 Equal Traffic Intensities”: Raw data for the analysis of decomposition error in tandem queues with equal traffic intensities, stored in two .csv-files (training and test data),

“02 Bottleneck Analyses”: Raw data for the analysis of decomposition error in tandem queues with bottleneck, stored in three .csv-files (downstream bottlenecks, upstream bottlenecks, and equal traffic intensities). The definition of all variables that appear in the data set is as follows:

1. EV Arrivals External: Expected value of the external arrival process
2. EV Service Upstream: Expected value of the upstream service process
3. EV Service Downstream: Expected value of the downstream service process
4. Utilization Upstream: Utilization of the upstream queue
5. Utilization Downstream: Utilization of the downstream queue
6. SCV Arrivals Downstream: Squared coefficient of variation of the downstream arrival process (that is, the upstream departure process)
7. SCV Service Upstream: Squared coefficient of variation of the upstream service process
8. SCV Service Downstream: Squared coefficient of variation of the downstream service process
9. EV Waiting Decomposition: Expected value of waiting time at the downstream queue, obtained with the discrete-time decomposition approach
10. EV Waiting Simulation: Expected value of waiting time at the downstream queue, obtained with discrete-event simulation
11. 95-perc Waiting Decomposition: 95th-percentile of waiting time at the downstream queue, obtained with the discrete-time decomposition approach
12. 95-perc Waiting Simulation: 95th-percentile of waiting time at the downstream queue, obtained with discrete-event simulation
13. EV Decomposition Error: Decomposition error with respect to the expected value of waiting time
14. 95-perc Decomposition Error: Decomposition error with respect to the 95th-percentile of waiting time

In this article, we present data and regression models for two tandem queue configurations [2]. First, we consider tandem queues with equal traffic intensity, and second, we present data for tandem queues with downstream bottlenecks. For both configurations, we present OLS regression and quantile regression to explain the variance of decomposition error with respect to the expected value and 95th-percentile of waiting time.

To this end, Table 1 specifies the variables used in the regression analyses. We use the squared coefficient of variation to describe the variability of the arrival and service processes and the utilization to describe the traffic intensity at the upstream and downstream queue, respectively.

**Table 1:** Specification of variables.

Variable	Description
scv(A_u), scv(A_d)	Squared coefficient of variation of the external (downstream) arrival process
scv(B_u), scv(B_d)	Squared coefficient of variation of the upstream (downstream) service process
rho_u, rho_d	Utilization parameters of the upstream (downstream) queue

## 2 Equal Traffic Intensities

This data set contains 1,166 data points that we partition into two subsets. The training data set consists of 932 randomly chosen data points, and the test data set consist of the remaining 234 data points.

**Fig. 1:**
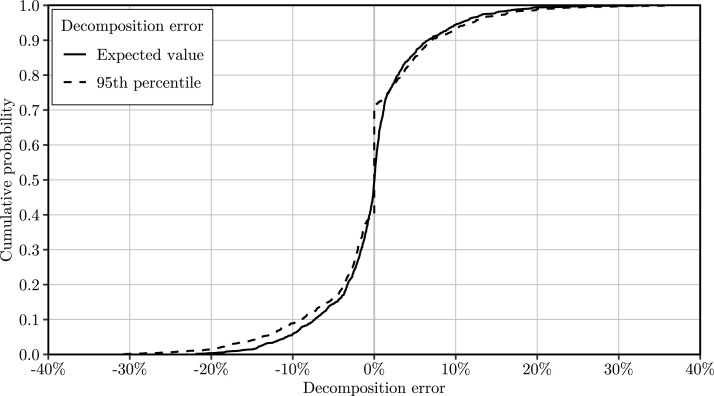
Empirical cumulative distribution functions of the decomposition error of waiting time regarding the expected value and 95th-percentile.

Fig. 1 shows the empirical cumulative distribution of decomposition error for the entire data set. It shows that the discrete-time decomposition approach both overestimates and underestimates waiting time in the same proportion. We find the relative errors in the range of −21.9% and 32.5% (referring to decomposition error with respect to the expected value) and −30.8% and 36.7% (referring to decomposition error with respect to the 95th-percentile). The mean absolute values of decomposition error equal 3.93% and 4.51% regarding decomposition error with respect to the expected value and the 95th-percentile of waiting time, respectively.

Table 2 provides the summarizing statistics for the IVs in the training data set and the flow parameters for the tandem queue. Note that the expected values for the service processes at the upstream and the downstream queue are equal, and thus, we list E(B) for both queues. We normalize the IVs of both subsets with the mean- and STD-values listed in Table 2.

**Table 2:** Summary statistics of the IVs and flow parameters in the training data set.

	Mean	STD	Min	Max
Rho	0.59	0.24	0.06	0.99
scv(B_u)	1.30	0.80	0.10	2.96
scv(B_d)	1.45	0.79	0.10	2.95
scv(A_d)	1.18	0.41	0.18	2.79

E(B)	12.32	6.35	1.23	29.0
E(A_d)	21.22	6.27	2.72	30.0

Table 3 presents the OLS and quantile regression coefficients and standard errors for decomposition error with respect to the expected value. The training data set was used to compute the coefficients. The OLS regression analysis is found to be statistically significant (F(10,921)=2123,p>.001), explaining the majority of the variance of the relative error of the expected value of waiting time ($R_{\text{Adj.}}^{2}=0.958$). The ANOVA reveals all direct effects and the majority of the interaction effects (with the exception of the interaction between downstream service time variability and utilization) to be statistically significant.

**Table 3:** OLS and quantile regression estimates for decomposition error (dependent variable is the expected value of waiting time) in tandem queues with equal traffic intensity.

	OLS	Q(.005)	Q(.025)	Q(.050)	Q(.950)	Q(.975)	Q(.995)
const.	0.0048***	-0.0068***	-0.0065***	-0.0039***	0.0228***	0.0309***	0.0361***
	0.0007	0.0012	0.0013	0.0011	0.0035	0.0034	0.0036
scv(B_u)	-0.0668***	-0.0460***	-0.0471***	-0.0551***	-0.0713***	-0.0770***	-0.0768***
	0.0017	0.0033	0.0046	0.0037	0.0084	0.0072	0.0074
scv(B_d)	-0.0039***	-0.0020***	-0.0011*	-0.0013**	-0.0081***	-0.0093***	-0.0076**
	0.0005	0.0006	0.0005	0.0005	0.0015	0.0015	0.0024
scv(A_d)	0.0591***	0.0437***	0.0424***	0.0530***	0.0585***	0.0676***	0.0731***
	0.0027	0.0056	0.007	0.0058	0.0129	0.0111	0.0108
Rho	-0.0325***	-0.0306***	-0.0301***	-0.0311***	-0.0300***	-0.0291***	-0.0283***
	0.0009	0.0014	0.0017	0.0015	0.0042	0.0041	0.0047
scv(B_u) × scv(B_d)	-0.0048***	-0.0087***	-0.0081***	-0.0064***	-0.0057***	-0.0052***	-0.0043*
	0.0008	0.0013	0.0016	0.0013	0.0013	0.0011	0.0019
scv(B_u) × scv(A_d)	0.0194***	0.0128***	0.0133***	0.0128***	0.0241***	0.0229***	0.0291***
	0.0005	0.0008	0.0007	0.0007	0.0033	0.0031	0.0054
scv(B_u) × rho	-0.0441***	-0.0293***	-0.0292***	-0.0366***	-0.0475***	-0.0488***	-0.0475***
	0.0011	0.0027	0.0033	0.0028	0.0052	0.0046	0.0058
scv(B_d) × scv(A_d)	0.0080***	0.0049***	0.0057***	0.0052***	0.0112***	0.0107***	0.0058
	0.0008	0.0014	0.0016	0.0012	0.0027	0.0025	0.0043
scv(B_d) × rho	-0.0006	0.0008	0.001	0.0017**	-0.0005	-0.0014	0.0006
	0.0005	0.0007	0.0008	0.0008	0.0018	0.0017	0.003
scv(A_d) × rho	-0.0405***	-0.0411***	-0.0396***	-0.0391***	-0.0407***	-0.0450***	-0.0518***
	0.0011	0.0023	0.0023	0.0019	0.0041	0.0043	0.0051

Adj. / Pseudo R^2^	0.958	0.917	0.902	0.895	0.829	0.843	0.872

Notes: standardized regression coefficients with standard errors listed below. The standard errors of quantile regression estimates are based on 100 bootstrapping replications. The sample is training data set with sample size 932.

*p < .1 **p < .05 ***p < .001.

The remaining columns of Table 3 present the coefficients of quantile regressions. The standard errors are obtained with 100 bootstrapping replications, respectively. The Pseudo R^2^ of each model is well above 0.8. All quantile regression equations show similar patterns of changes in coefficient values as the OLS regression. We find the majority of direct and interaction effects to be statistically significant. As in the OLS regression, the interaction effect between the service process variability (at the upstream queueing system) and the utilization is found to be non-significant among each model. While the absolute sizes of the coefficients for most factors vary little across the equations, it should be noted that the weights of the service process variability at the upstream queueing system, and the arrival process variability at the downstream queueing system rise with increasing quantile.

Table 4 presents the OLS regression coefficients for decomposition error with respect to the 95th-percentile of waiting time. We find a statistically significant OLS regression equation (F(10,921)=1064,p>.001), which explains the majority of the variance ($R_{\text{Adj.}}^{2}=0.920$) of decomposition error. All direct effects are statistically significant. The impact patterns of the interaction effects are the same as in Table 3.

**Table 4:** OLS and quantile regression estimates for decomposition error (dependent variable is the 95th-percentile of waiting time) in tandem queues with equal traffic intensity.

	OLS	Q(.005)	Q(.025)	Q(.050)	Q(.950)	Q(.975)	Q(.995)
const.	0.0052***	-0.0169***	-0.0107***	-0.0093***	0.0289***	0.0349***	0.0838***
	0.0012	0.0023	0.0022	0.002	0.0033	0.0064	0.0163
scv(B_u)	-0.0735***	-0.0671***	-0.0631***	-0.0547***	-0.0847***	-0.0931***	-0.1010***
	0.0028	0.0082	0.0089	0.0074	0.0109	0.0129	0.0146
scv(B_d)	-0.0046***	-0.0036	0.0002	0.0004	-0.0093***	-0.0120***	-0.0413***
	0.0008	0.0031	0.0021	0.0012	0.0019	0.0044	0.0097
scv(A_d)	0.0680***	0.0916***	0.0776***	0.0554***	0.0735***	0.0777***	0.0591***
	0.0046	0.0146	0.0163	0.0116	0.0169	0.0198	0.0236
Rho	-0.0381***	-0.0351***	-0.0360***	-0.0321***	-0.0439***	-0.0471***	-0.0636***
	0.0015	0.0029	0.0031	0.0022	0.0052	0.0071	0.012
scv(B_u) × scv(B_d)	-0.0038***	-0.0134*	-0.0052**	-0.0066***	-0.0095**	-0.0035	0.0254**
	0.0013	0.0062	0.0019	0.0014	0.0032	0.0069	0.0114
scv(B_u) × scv(A_d)	0.0189***	0.0078***	0.0091***	0.0113***	0.0241***	0.0322***	0.0263***
	0.0008	0.0012	0.0015	0.0013	0.003	0.005	0.0073
scv(B_u) × rho	-0.0476***	-0.0291***	-0.0378***	-0.0338***	-0.0649***	-0.0676***	-0.0342***
	0.0019	0.0086	0.0067	0.0053	0.0072	0.0097	0.0128
scv(B_d) × scv(A_d)	0.0092***	0.0089	0.0005	0.0049*	0.0148***	0.009	-0.0046
	0.0014	0.0063	0.0032	0.0019	0.0039	0.0058	0.0081
scv(B_d) × rho	0.0008	0.0109***	0.0056*	0.0030*	0.0038	0.0065	0.0218***
	0.0008	0.0028	0.0022	0.0015	0.0026	0.0045	0.0071
scv(A_d) × rho	-0.0522***	-0.0740***	-0.0597***	-0.0520***	-0.0468***	-0.0476***	-0.0503***
	0.0019	0.0061	0.0055	0.0029	0.0048	0.006	0.011

Adj. / Pseudo R^2^	0.920	0.809	0.798	0.807	0.698	0.681	0.635

Notes: standardized regression coefficients with standard errors listed below. The standard errors of quantile regression estimates are based on 100 bootstrapping replications. The sample is training data set with sample size 932.

*p < .1 **p < .05 ***p < .001.

The remaining columns of Table 4 show the regression coefficients of quantile regressions. The standard errors are computed with 100 bootstrapping replications, respectively, with Pseudo R^2^ of all models well above 0.6. Except for the service process variability at the downstream queueing system, which is non-significant for the models with τ≤.05, all direct effects are found to be statistically significant among each regression model. The majority of interaction coefficients is found to be significant or marginally significant. However, we did find non-significant coefficients among the interaction effect of the service process variability and the arrival process variability (both at the downstream queueing system), as well as in the Q(.975) model. As in Table 3, the absolute sizes of coefficients vary little for most factors across the equations. However, the weight of the utilization increases by rising quantiles, while (in contrast to Table 2) the weight of the arrival process variability decreases.

## 3 Downstream bottlenecks

As Suresh and Witt [3] mention, in a narrower sense, the bottleneck is the queue with the highest traffic intensity. However, increasing the traffic intensity of a queue by only a small amount may shift the bottleneck position. Therefore, it is intuitive to state that either of the queues is the bottleneck if it's utilization is substantially greater than some ε, $|\rho_{u}-\rho_{d}|>\varepsilon$.

We created a data set that contains 969 data points and choose ε=0.1 to split the data into three subsets. In the first data set (403 data points), the downstream queue is the bottleneck, in the second data set (131 data points), the traffic intensities are similar ($|\rho_{u}-\rho_{d}|\leq \varepsilon$), and in the third data set (435 data points), the upstream queue is the bottleneck.

Analogous to the analyses of tandem queues with equal traffic intensities, we use OLS and quantile regression to model decomposition error with respect to the expected value and 95th-percentile of waiting time. We use the first and the second data set to compute the regression coefficients. Table 5 and Table 6 show the results with respect to the expected value and the 95th-percentile of waiting time, respectively.

**Table 5:** OLS and quantile regression estimates for decomposition error (dependent variable is the expected value of waiting time) in tandem queues with downstream bottlenecks (cont. on next page).

	OLS	Q(.005)	Q(.025)	Q(.050)	Q(.950)	Q(.975)	Q(.995)
const.	-0.0704***	-0.2019***	-0.1530***	-0.1332***	-0.0426**	-0.0184	-0.0184
	0.0072	0.0492	0.0359	0.0266	0.013	0.0176	0.0211
scv(B_u)	0.1314***	0.1222	0.2307**	0.2125*	0.1324**	0.0767	0.1116
	0.0233	0.1615	0.0989	0.072	0.0411	0.0542	0.0708
scv(B_d)	0.0082***	-0.021	0.0079	0.0099*	0.0059	0.0057	0.0052
	0.0015	0.023	0.0116	0.0051	0.0036	0.0041	0.0044
scv(A_d)	-0.3605***	-0.4273	-0.5899**	-0.5556***	-0.3532***	-0.2373**	-0.2985**
	0.0473	0.3194	0.1963	0.1492	0.0847	0.1126	0.1434
rho_u	0.1000***	0.1741*	0.1725**	0.1558**	0.0964***	0.0680**	0.0848**
	0.0145	0.0961	0.0619	0.0477	0.0249	0.0321	0.0407
rho_d	-0.0255***	-0.0738***	-0.0442***	-0.0361***	-0.0090**	-0.0098**	-0.0036
	0.002	0.0121	0.0094	0.0055	0.004	0.0048	0.0049
scv(B_u) × scv(B_d)	0.0019	-0.0319*	-0.0087	-0.0071**	0.0054**	0.0075**	0.0056*
	0.0022	0.0182	0.0127	0.0035	0.002	0.0026	0.0032
scv(B_u) × scv(A_d)	0.0097***	0.0071	0.0006	0.0009	0.0259***	0.0261***	0.0242***
	0.0018	0.0087	0.0074	0.0049	0.0064	0.0062	0.0063
scv(B_u) × rho_u	0.1006***	0.1671	0.1721**	0.1668**	0.0914**	0.0486	0.0786
	0.0189	0.1234	0.0694	0.0546	0.0324	0.042	0.0551
scv(B_u) × rho_d	0.0045*	-0.0202	0.0058	0.0004	0.0071***	0.0059**	0.0049*
	0.002	0.0208	0.0138	0.0056	0.0018	0.0021	0.0025
scv(B_d) × scv(A_d)	0.0142***	0.0329**	0.0229**	0.0204**	0.0028	0.0005	0.0037
	0.0024	0.0159	0.0103	0.0067	0.006	0.0066	0.0067
scv(B_d) × rho_u	-0.0058**	0.0313	0.0026	0.0009	-0.0061*	-0.0057	-0.0064
	0.0018	0.0192	0.0111	0.0044	0.0033	0.0036	0.0043
scv(B_d) × rho_d	0.0091***	-0.0107	0.002	0.0039	0.0082***	0.0064***	0.0059***
	0.0017	0.0147	0.0079	0.003	0.0014	0.0014	0.0017
scv(A_d) × rho_u	0.1022***	0.0953	0.1661**	0.1548***	0.0931***	0.0675**	0.0743**
	0.0108	0.0784	0.0518	0.0383	0.0202	0.0265	0.0314
scv(A_d) × rho_d	-0.0712***	-0.0356	-0.0700***	-0.0647***	-0.0643***	-0.0673***	-0.0584***
	0.0044	0.0281	0.0192	0.0133	0.0109	0.0115	0.012
rho_u × rho_d	0.0008	-0.008	-0.0038	-0.0048*	0.0097**	0.0107**	0.0117**
	0.0023	0.0053	0.0042	0.0028	0.0036	0.0034	0.0042

Adj. / Pseudo R^2^	0.833	0.698	0.687	0.717	0.715	0.760	0.824

Notes: standardized regression coefficients with standard errors listed below. The standard errors of quantile regression estimates are based on 100 bootstrapping replications. The sample size is 534.

*p < .1 **p < .05 ***p < .001.

**Table 6:** OLS and quantile regression estimates for decomposition error (dependent variable is the 95th-percentile of waiting time) in tandem queues with downstream bottlenecks (cont. on next page).

	OLS	Q(.005)	Q(.025)	Q(.050)	Q(.950)	Q(.975)	Q(.995)
const.	-0.0831***	-0.2828***	-0.2213***	-0.1591***	-0.0492**	-0.0275	-0.0156
	0.0087	0.0695	0.056	0.0414	0.0149	0.0173	0.0186
scv(B_u)	0.1549***	0.2868	0.3651**	0.2204*	0.1474**	0.0941*	0.0841
	0.0281	0.2243	0.1577	0.1132	0.0479	0.0549	0.058
scv(B_d)	0.0101***	-0.0162	0.0099	0.0097	0.0033	0.0041	0.0056
	0.0018	0.025	0.0137	0.0074	0.0041	0.0039	0.0043
scv(A_d)	-0.4181***	-0.7720*	-0.8768**	-0.5957**	-0.3892***	-0.2792**	-0.2494**
	0.0569	0.4493	0.3167	0.2368	0.1006	0.1145	0.1197
rho_u	0.1184***	0.2926**	0.2665**	0.1663**	0.1078***	0.0805**	0.0773**
	0.0175	0.141	0.1032	0.0751	0.0301	0.0333	0.0343
rho_d	-0.0322***	-0.0818***	-0.0535***	-0.0457***	-0.0117**	-0.0146**	-0.0165**
	0.0025	0.013	0.0107	0.0084	0.0043	0.0044	0.0055
scv(B_u) × scv(B_d)	0.0014	-0.0181	-0.0086	-0.0085	0.0068**	0.0060**	0.0075**
	0.0026	0.0182	0.0152	0.0057	0.0025	0.0028	0.0032
scv(B_u) × scv(A_d)	0.0068**	0.0046	0.0004	-0.0015	0.0225***	0.0218**	0.0215**
	0.0022	0.0115	0.0086	0.0068	0.0065	0.0067	0.0071
scv(B_u) × rho_u	0.1202***	0.3086*	0.2814**	0.1779**	0.1013**	0.0594	0.0535
	0.0227	0.1856	0.1211	0.0827	0.0368	0.0421	0.0441
scv(B_u) × rho_d	0.0053*	-0.0228	0.0085	-0.0025	0.0064**	0.0077***	0.0074**
	0.0025	0.0236	0.0185	0.0091	0.002	0.002	0.0025
scv(B_d) × scv(A_d)	0.0197***	0.0287*	0.0254**	0.0273***	0.0069	0.0071	0.0064
	0.0029	0.0166	0.0114	0.007	0.0061	0.0061	0.0082
scv(B_d) × rho_u	-0.0054*	0.0334	0.0054	-0.0008	-0.0083**	-0.0074**	-0.0055
	0.0022	0.0243	0.015	0.0068	0.0027	0.0031	0.0039
scv(B_d) × rho_d	0.0087***	-0.012	-0.001	0.0041	0.0060***	0.0052***	0.0056**
	0.0021	0.0168	0.0097	0.0045	0.0016	0.0016	0.0018
scv(A_d) × rho_u	0.1162***	0.1625	0.2289**	0.1711**	0.1048***	0.0827**	0.0728**
	0.013	0.0987	0.0789	0.0646	0.0256	0.0278	0.0295
scv(A_d) × rho_d	-0.0830***	-0.0385	-0.0854**	-0.0841***	-0.0675***	-0.0740***	-0.0761***
	0.0052	0.0354	0.029	0.0209	0.0096	0.0091	0.0111
rho_u × rho_d	-0.0001	-0.0077	-0.0047	-0.0022	0.0091**	0.0082**	0.0074*
	0.0028	0.0075	0.0061	0.005	0.003	0.0031	0.0038

Adj. / Pseudo R^2^	0.826	0.637	0.602	0.626	0.703	0.753	0.822

Notes: standardized regression coefficients with standard errors listed below. The standard errors of quantile regression estimates are based on 100 bootstrapping replications. The sample size is 534.

*p < .1 **p < .05 ***p < .001.

## 4 Experimental Design, Materials and Methods

We use the algorithm described in [4] to generate 1,166 data points in a four-dimensional space-filling latin hypercube design (data sets with equal traffic intensities), and 969 data points in a five-dimensional space-filling latin hypercube design (data sets for bottleneck analyses). The expected values of the external inter-arrival and the service times are independently randomly selected from the interval [1.0, 30.0]. To create the data points with equal traffic intensity, the expected values of service time are equal for the upstream and the downstream queue. The variability parameters of the service time distributions are independently randomly selected from the interval [0.1, 3.0].

In our analyses [1] we assume that the random variables describing the service processes are described by discretized gamma distributions. Let X be a gamma-distributed random variable with shape parameter k and scale parameter θ. The probability density function of X is given by [1,5]

$$f\left(x;k,\theta \right)=\frac{x^{k-1}e^{-x/\theta }}{\theta^{k}\Gamma \left(k\right)},\;x,k,\theta >0,$$

where Γ(k) is the gamma function. In order to generate gamma-distributed random variables X with predefined values for E(X) and scv(X), we use the well-known closed-form expressions for the shape and scale parameters of the gamma function [1,5],

$$E\left(X\right)=k\theta ,\;Var\left(X\right)=k\theta^{2}.$$

We use the squared coefficient of variation (scv) as normalized measure of statistical dispersion to measure the process variability. Let E(X) define the expected value of, and Var(X) its variance. The variability of X is defined as [1]

$$scv\left(X\right)=Var\left(X\right)/E^{2}\left(X\right).$$

## Ethics Statements

The authors declare that they have no conflict of interests.

## CRediT authorship contribution statement

**Christoph Jacobi:** Conceptualization, Methodology, Software, Formal analysis, Visualization, Writing – original draft, Writing – review & editing. **Kai Furmans:** Conceptualization, Supervision.

## Declaration of Competing Interest

The authors declare that they have no known competing financial interests or personal relationships that could have appeared to influence the work reported in this paper.

## Data Availability

Data sets for the analysis of decomposition error in discrete-time open tandem queues (Original data) (KITopen Repository).
